# Antidiabetic Property of *Symplocos cochinchinensis* Is Mediated by Inhibition of Alpha Glucosidase and Enhanced Insulin Sensitivity

**DOI:** 10.1371/journal.pone.0105829

**Published:** 2014-09-03

**Authors:** Kalathookunnel Antony Antu, Mariam Philip Riya, Arvind Mishra, Karunakaran S. Anilkumar, Chandrasekharan K. Chandrakanth, Akhilesh K. Tamrakar, Arvind K. Srivastava, K. Gopalan Raghu

**Affiliations:** 1 Agroprocessing and Natural Products Division, Council of Scientific and Industrial Research-National Institute for Interdisciplinary Science and Technology (CSIR-NIIST), Thiruvananthapuram, Kerala, India; 2 Division of Biochemistry, Council of Scientific and Industrial Research-Central Drug Research Institute (CSIR-CDRI), Lucknow, Uttar Pradesh, India; 3 Medicinal Chemistry Division, CSIR-CDRI, Lucknow, Uttar Pradesh, India; 4 Division of Pharmacology, CSIR-CDRI, Lucknow, Uttar Pradesh, India; Kobe University, Japan

## Abstract

The study is designed to find out the biochemical basis of antidiabetic property of *Symplocos cochinchinensis* (SC), the main ingredient of ‘*Nisakathakadi*’ an *Ayurvedic* decoction for diabetes. Since diabetes is a multifactorial disease, ethanolic extract of the bark (SCE) and its fractions (hexane, dichloromethane, ethyl acetate and 90% ethanol) were evaluated by *in vitro* methods against multiple targets relevant to diabetes such as the alpha glucosidase inhibition, glucose uptake, adipogenic potential, oxidative stress, pancreatic beta cell proliferation, inhibition of protein glycation, protein tyrosine phosphatase-1B (PTP-1B) and dipeptidyl peptidase-IV (DPP-IV). Among the extracts, SCE exhibited comparatively better activity like alpha glucosidase inhibition (IC_50_ value-82.07±2.10 µg/mL), insulin dependent glucose uptake (3 fold increase) in L6 myotubes, pancreatic beta cell regeneration in RIN-m5F (3.5 fold increase) and reduced triglyceride accumulation (22% decrease) in 3T3L1 cells, protection from hyperglycemia induced generation of reactive oxygen species in HepG2 cells (59.57% decrease) with moderate antiglycation and PTP-1B inhibition. Chemical characterization by HPLC revealed the superiority of SCE over other extracts due to presence and quantity of bioactives (beta-sitosterol, phloretin 2′glucoside, oleanolic acid) in addition to minerals like magnesium, calcium, potassium, sodium, zinc and manganese. So SCE has been subjected to oral sucrose tolerance test to evaluate its antihyperglycemic property in mild diabetic and diabetic animal models. SCE showed significant antihyperglycemic activity in *in vivo* diabetic models. We conclude that SC mediates the antidiabetic activity mainly via alpha glucosidase inhibition, improved insulin sensitivity, with moderate antiglycation and antioxidant activity.

## Introduction

Diabetes mellitus is a global health threat associated with increased morbidity, mortality and poor quality of life which is characterized by chronic hyperglycemia [1]. Hyperglycemia leads to vascular complications via glucose toxicity and oxidative stress [2] and its proper control is an important therapeutic strategy to prevent diabetic complications [3]. Major determinants of postprandial hyperglycemic variations include gut digestion and absorption rate, available insulin response and tissue insulin sensitivity [4]. A medication that can address these abnormalities along with oxidative stress may be quite beneficial to diabetes. Current therapies include insulin and various oral agents such as sulfonylureas, biguanides, alpha-glucosidase inhibitors and gliptins, which are used as monotherapy or in combination to achieve better glycemic regulation [3]. These medications have some undesirable effects [5] and managing diabetes without side effects is still being a challenge. Hence the search for more effective and safer therapeutic agents of natural origin has been found to be valuable.

Traditional medicines are frequently used in urban settings as an alternative in daily healthcare and it recommends complex herbal mixtures and multi-compound extracts [6]. Synergistic properties of herbal medicines due to the presence of variety of components within a single herbal extract are beneficial to multifactorial diseases like diabetes [7]. Herbal medicines have played an important role in treating diabetes in various parts of the world for centuries. *Ayurveda*, a system of traditional medicine native to Indian subcontinent always plays major role in primary health care of both rural and urban populations of India [8]. *Symplocos cochinchinensis* (Lour.) S. Moore. (SC) from the family Symplocaceae, is a medicinal plant with anti-inflammatory, antitumor, antimicrobial and antidiabetic properties [9], [10]. The bark of SC is one of the key ingredients of *Nisakathakadi Kashayam* (decoction); a very effective *Ayurvedic* preparation for diabetes mentioned in the ancient script ‘*Sahasrayogam*’ [11]. For wider acceptability of the health benefits of SC, a detailed scientific investigation on its mode of action on various biochemical targets relevant to diabetes is mandatory. But any thorough study illustrating the mechanism of action of SC or its biochemical targets relevant to diabetes is not available in literature. Here, attempts are made to see the main bioactives responsible for its antidiabetic property and to elucidate the mode of action of SC using selected biochemical targets relevant to diabetes.

## Materials and Methods

### Chemicals and Reagents

Streptozotocin (≥98%), 2,2 diphenyl-1-1-picryl hydrazyl (DPPH), 4-nitro phenyl alpha-D- glucopyranoside, yeast alpha-glucosidase, acarbose, gallic acid, tannic acid, quercetin, trolox, diprotin A, suramin, beta-sitosterol, phloretin 2′glucoside, oleanolic acid, rosiglitazone, metformin, cytochalasin B, 2-deoxyglucose, 3-isobutyl-1-methylxanthine (IBMX), dexamethasone, insulin, dimethyl sulphoxide (DMSO) and all other chemicals and biochemicals unless otherwise noted were from Sigma (St. Louis, MO, USA). 2-deoxy-d-[^3^H]-glucose (2-DG) was from GE Healthcare, UK. All the positive controls used were of HPLC grade.

### Ethics statement

No specific permission was required for the collection of the plant material. This plant is plenty available in this specific area (Palode, Thiruvanathapuram) and there is no restriction for the collection of the plant. It is not an endangered or protected species. The location is not privately-owned or protected in any way. According to the guidelines of the Committee for the Purpose of Control and Supervision of Experiments on Animals (CPCSEA) formed by the Government of India in 1964, proper sanction had been obtained for animal experiments from CSIR-CDRI institutional animal ethics committee (Ethics Committee Approval Reference No. IAEC/2008/63/Renewal 04 dated 16.05.2012). Approval was obtained specifically for the animal experiments of this study from CSIR-CDRI institutional animal ethics committee. Animals were sacrificed by cervical dislocation under light ether anaesthesia as per ethics committee guidelines.

### Plant material

The bark of SC was collected from Palode, Thiruvananthapuram (8°29′N, 76°59′E) during July 2011 and authenticated by Dr. Biju Haridas, Taxonomist, Jawaharlal Nehru Tropical Botanic Garden and Research Institute (JNTBGRI), Thiruvananthapuram, Kerala. A voucher specimen (No. 66498) was stored at the herbarium of JNTBGRI. 2 kg dry powder was extracted by maceration at 35–37°C; five times for 18 to 20 hrs with 70% ethanol [12]. Then it was filtered under vacuum and dried using rotary evaporator (Heidolph, Schwabach, Germany) at 35–40°C. This *Symplocos cochinchinensis* hydroethanol extract was designated as SCE. SCE was fractionated using 4 different solvents based on polarity; n-hexane (SCH), dichloromethane (SCD), ethyl acetate (SCEC) & 90% ethyl alcohol (SCEL). The SCE and its fractions were stored at 4°C, protected from light and humidity.

### HPLC analysis

The HPLC analysis was carried out as described previously with slight modifications [13] on LC-20AD HPLC system (Shimadzu, Tokyo, Japan) equipped with the PDA detector, SPD-M20A and LC solutions software. The chromatographic separations were performed using Phenomenex Luna C-18 Column (150 mm×4.6 mm I. D, 5 µm), with a flow rate of 0.5 mL/min and a sample injection volume of 20 µL. The mobile phase used was acetonitrile (A) and water (B) with an isocratic elution ratio of 85∶15 (A∶B (v/v)) in 20 min. The sample was monitored with UV detection at 210 nm at 40°C.

### Atomic Absorption Spectrophotometer (AAS) analysis

SCE (25 mg/mL) was digested in dilute HCl (7∶3). The concentration of minerals was quantified (mg/g of sample) by atomic absorption spectrophotometer (Perkin Elmer Inc. USA).

### Quantification of Total Phenolic Content (TPC), Total Tannin Content (TTC) and Total Flavonoid Content (TFC)

TPC was determined as described previously [14], and were expressed as milligram gallic acid equivalents per gram of extract (mg GAE/g). Tannin estimation was done by the indirect method [15]. TTC was expressed as milligram tannic acid equivalents per gram of extract (mg TAE/g). TFC estimation was done as described previously [16] and expressed as milligram quercetin equivalents per gram of extract (mg QE/g).

### *In vitro* alpha glucosidase (AG), dipeptidyl peptidase-IV (DPP-IV) & protein tyrosine phosphatase-1B (PTP-1B) inhibition assay

Yeast and rat intestinal AG (EC 3.2.1.20) inhibitory property of the extracts were determined as described previously [17] using acarbose as standard. All the extracts were checked for DPP-IV (EC 3.4.14.5) inhibition using the kit from Cayman chemicals (Ann Arbor, MI, USA). Diprotin A was used as the standard. PTP-1B (EC 3.3.3.48) inhibitory property of extracts was evaluated using the kit from Calbiochem (Darmstadt, Germany). Percentage inhibition values were plotted against the corresponding concentrations of the sample to obtain IC_50_ value.

### Determination of antioxidant potential and metal chelation activity

The antioxidant activity of extracts was assessed by DPPH method [18] with gallic acid as standard. 2,2′-azino-bis(3-ethylbenzothiazoline-6-sulphonic acid) (ABTS) radical scavenging activity was determined using assay kit (Zen-Bio Inc., NC, USA) and trolox was the standard. The hydroxyl radical scavenging activity was measured by the deoxyribose method [19] with catechin as standard. The chelation of ferrous ions by the extracts was estimated using ferrozine method [20] and EDTA was used as the standard. IC_50_ values were calculated and compared with the respective standards.

### Determination of antiglycation activity

Advanced glycation end products (AGEs) derived from bovine serum albumin (BSA) were quantified using the previous method [21]. BSA in the presence of ribose in phosphate buffered saline was served as control. AGE fluorescence (λ_ex_370 nm; λ_em_ 440 nm) was measured in terms of relative fluorescence unit (RFU) after 24 h and 7 days of incubation. Investigations after 24 h and 7 days incubation are designated as day1 and day7 experiments respectively for future references. The data was compared with the reference compound quercetin (100 µM). AGEs formed were also processed for complexity analysis to check whether test material has capacity to block the formation of glycated products [21] using scanning electron microscope (SEM; Carl Zeiss, Munich, Germany).

### Cell culture

HepG2 and L6 cell lines were obtained from National Centre for Cell Science, Pune, India. The HepG2 cells were maintained in low glucose (5.5 mM) DMEM supplemented with 10% FBS and 1% antibiotic/antimycotic solution (10,000 U/mL penicillin G, 10 mg/mL streptomycin, 25 µg/mL amphotericin B), with 5% CO_2_ at 37°C. L6 skeletal muscle cells were maintained in alpha-MEM supplemented with 10% FBS and 1% antibiotic/antimycotic solution at 5% CO_2_ at 37°C. Differentiation was induced by switching confluent cells to medium supplemented with 2% FBS. Experiments were performed in differentiated myotubes. RIN-m5F cells (ATCC, USA) were cultured in RPMI-1640 supplemented with 10% FBS and 1% antibiotic/antimycotic solution at 5% CO_2_ at 37°C. MIN-6 cells (ATCC, USA) were maintained in DMEM supplemented with 10% FBS, 1% antibiotic/antimycotic solution, 100 µg/mL L-glutamine, 10 µL/L beta - mercaptoethanol at 5% CO_2_ at 37°C. 3T3-L1 murine preadipocytes (ATCC, USA) were cultured in DMEM supplemented with 10% FBS and antibiotics. Differentiation was induced by switching to DMEM with 500 µM 3-isobutyl-1-methylxanthine (IBMX), 10 µM dexamethasone and 500 nM insulin (MDI) for 48 h. Differentiation was then maintained in DMEM containing 10% FBS and 500 nM insulin for 8 days.

### Determination of cell viability

The extracts were dissolved in DMSO for application to cell cultures and final concentration of DMSO was fixed at 0.1% for all cell based assays. The cytotoxicity was checked by MTT assay kit (Cayman chemicals, Ann Arbor, MI, USA). HepG2, RIN-m5F, MIN-6, L6 and 3T3-L1 cells were seeded at a density of 4×10^4^ cells/well in 24 well plate and incubated for 24 h. Cells were treated with various concentrations of extract and incubated for 24 h. Then, cell viability in HepG2, L6 and 3T3-L1 or proliferation in RIN-m5F and MIN-6 was evaluated.

### Hyperglycemia induced oxidative stress

The cells were maintained in low glucose medium (5.5 mM) for the initial 24 h, then switched over to high glucose (25 mM) medium with or without the extracts or quercetin (positive control) to check whether test material prevent the generation of oxidative stress. The intracellular reactive oxygen species (ROS) production was monitored with the fluorescent probe CM-H_2_DCFDA [22].

### Glucose uptake

The 2-deoxy glucose uptake in L6 myotubes was performed as described previously [23]. Glucose uptake measured in triplicate and normalized to total protein, was expressed as fold induction with respect to unstimulated cells. Rosiglitazone and metformin were the standards.

### Adipocyte differentiation

The adipogenic potential of all the extracts (30 µg/mL) was assessed in 3T3-L1 preadipocyte over untreated cells by quantifying the accumulation of triglycerides using oil red O staining on day 8 [24], [25]. Rosiglitazone was used as standard. The cell lysate from all experimental groups was prepared according to the previous method [21] and assayed for GPDH (EC 1.1.1.8) activity using a Takara GPDH Assay Kit (Takara Bio Inc, Otsu, Japan). The membrane fraction for DGAT1 assay was collected as described previously [26]. DGAT1 activity was measured using the kit from MyBioSourse.com (San Diego, CA, USA). Total cellular TG was extracted as reported previously [27]. TG content was assayed using a TG assay kit (Cayman Chemicals). The protein content was measured and normalized for GPDH, DGAT 1 and TG assays using a bicinchoninic acid kit (Pierce, Rockford, IL USA). The adiponectin level in the residual media was measured using adiponectin assay kit (Cayman Chemicals).

### Animals

Male albino rats of Sprague Dawley (SD) strain (7–8 weeks old, 160±20 g), bred at animal facility of CSIR-CDRI, Lucknow were selected for this study. Rats were housed in polypropylene cages (5 rats per cage) under an ambient temperature of 23±2°C; 50–60% relative humidity; light 300 lux at floor level with regular 12 h light/dark cycle. Animals were maintained on a standard pellet diet and water *ad libitum*.

### Oral Sucrose Tolerance Test (OSTT) in normal rats

For this normal SD rats were fasted for 16 h. Animals showing fasting blood glucose level (BGL) between 70 to 90 mg/dL were divided into 6 groups containing 6 animals each. Animals of experimental groups were orally administered SCE (100, 250 and 500 mg/kg body weight (bw)), metformin (100 mg/kg bw) or acarbose (50 mg/kg bw) dissolved in 1.0% gum acacia. The dose of SCE was selected on the basis of dosage of ‘*Nisakathakadi Kashayam*’ for human use. 10–15 mL of this preparation containing approximately 3.5 g of SC bark including other 7 herbs in equal amount, thrice in a day is generally prescribed for patients [11]. Ethanol extract has been used for *in vivo* study due to the yield of more bioactive molecules and less toxicity of the solvent [28]. Since its selective nature, 70% ethanol is the most suitable solvent for *in vivo* pharmacological evaluation compared to other solvents; it will dissolve only the required bioactive constituents with minimum amount of the inert materials [28]. Animals of control group were given an equal volume of 1.0% gum acacia. Rats were loaded with sucrose (10 g/kg bw) orally 30 min after administration of test sample or vehicle. BGL was estimated at 30, 60, 90 and 120 min post administration. Food but not water was withheld during the course of experimentation [29].

### OSTT in sucrose loaded mild diabetic rat model (SLM)

Animals were made diabetic by injecting streptozotocin (60 mg/kg in 100 mM citrate buffer-pH 4.5) intraperitoneally after overnight fasting. Animals showing fasting BGL<200 mg/dL after 72 h were selected, termed as mild diabetic [30] and divided into 4 groups of 6 animals each. Animals of experimental group were administered SCE (500 mg/kg bw), metformin (100 mg/kg bw) or acarbose (50 mg/kg bw). Mild diabetic control group were given an equal amount of 1.0% gum acacia. A sucrose load (10 g/kg) was given to each animal orally 30 min after test sample or vehicle. BGL was determined at 30, 60, 90 and 120 min post-administration of sucrose [29].

### OSTT in sucrose-challenged streptozotocin-diabetic rat model (STZ-S)

Like SLM, rats were made diabetic. Animals of BGL>350 mg/dL after 72 h were selected, termed as diabetic [30], and divided into 4 groups of 6 animals each. Experimental groups were administered SCE, metformin or acarbose like SLM. Diabetic control group received equal amount of 1.0% gum acacia. Rats were loaded with sucrose (3 g/kg bw) orally 30 min after test sample or vehicle. BGL was checked at 30, 60, 90, 120, 180, 240, 300 and 1440 min (24 h), respectively [29]. Acarbose has been selected as one of the positive control as it is the alpha-glucosidase inhibitor which can improve long term glycemic control in patients with diabetes [31]. Metformin is the widely used antidiabetic to treat the cardinal symptoms of diabetes like polyphagia, polydipsia, polyuria and insulin resistance due to its pleiotropic effect via various targets and it shows wide tolerance and less toxicity compared to other antidiabetics [32]. Due to the wider acceptability of metformin as an antidiabetic drug, we used it as a positive control.

### Statistical analysis

Quantitative glucose tolerance of each group was calculated by the area under the curve (AUC) method using GraphPad Prism software version 3 (GraphPad Software Inc., La Jolla, CA, USA). All other results were analyzed using a statistical program SPSS/PC+, version 11.0 (SPSS Inc., Chicago, IL, USA). Data are presented as mean ± SD, from 3 independent experiments with triplicates. P≤0.05 was considered to be significant.

## Results

### Phytochemical characterization

HPLC analysis confirmed the presence of beta-sitosterol (111.62±4.12 mg/g), phloretin 2′glucoside (98.32±4.87 mg/g) and oleanolic acid (63.89±3.03 mg/g) in *Symplocos cochinchinensis* ethanolic extract (SCE); phloretin 2′glucoside (508.46±11.63 mg/g) and oleanolic acid (39.09±1.73 mg/g) in ethyl acetate fraction of SCE (SCEC); beta-sitosterol (145.56±4.63 mg/g) in hexane fraction of SCE (SCH); beta-sitosterol (152.29±6.31 mg/g) and phloretin 2′glucoside (188.97±6.41 mg/g) in dichloromethane fraction of SCE (SCD); phloretin 2′glucoside (273.65±7.63 mg g−1) in ethyl acetate fraction of SCE (SCEL) (Fig. S1 and S2 in Supporting Information S1) [33]. Analysis of minerals by AAS for micro-nutrients revealed presence of various minerals like zinc (0.014±0.0005 mg/g) manganese (0.096±0.0041 mg/g), iron (0.147±0.005 mg/g), sodium (1.387±0.062 mg/g), potassium (2.496±0.11 mg/g), magnesium (4.368±0.203 mg/g) and calcium (46.799±2.15 mg/g). The dry yield, TPC, TTC and TFC of the extracts were shown in Table 1. Since SCE exhibited comparatively better activity with respect to various *in vitro* targets and its high content of bioactives, SCE was taken forward for *in vivo* study. Moreover, in Indian traditional system of medicine (*Ayurveda*) most of the decoctions are hydroalcohol based (eg. *Arishta* and *Kashaya*).

**Table 1:** Dry yield, Total Phenolic Content (TPC), Total tannin Content (TTC) and Total Flavonoid Content (TFC) of test materials.

Sl no.	Sample	Dry yield as % weight of dry plant material	Total phenolic content (TPC) in mg GAE/g	Total flavonoid content (TFC) in mg QE/g	Total tannin content (TTC) in in mg TAE/g
1	SCE	12.35	53.72	19.35	10.47
2	SCH	0.50	13.40	8.56	-
3	SCD	0.32	36.27	19.85	-
4	SCEC	0.55	57.28	26.35	17.54
5	SCEL	2.91	54.68	22.85	13.26

### *In vitro* AG, DPP-IV and PTP1-B inhibitory property

**Figure 1:**
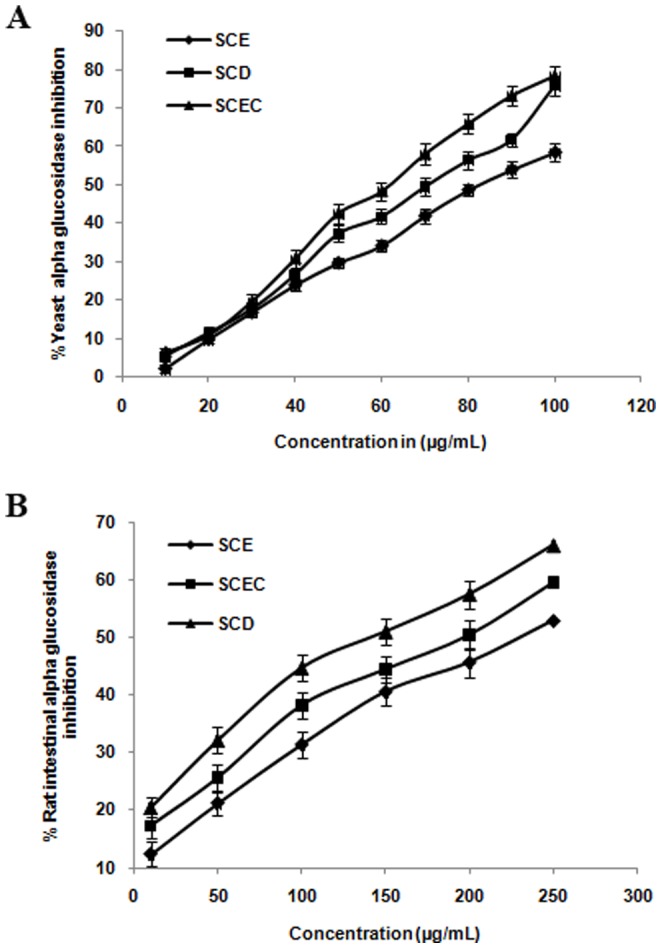
SCE SCD and SCEC exhibited alpha-glucosidase inhibitory property. (A) Yeast alpha glucosidase inhibition. (B) Rat intestinal alpha glucosidase inhibition. Values are means ± SD; n = 3. SCE, *S. cochinchinensis* (SC) ethanol extract; SCD, SC dichloromethane fraction and SCEC, SC ethyl acetate fraction.

**Figure 2:**
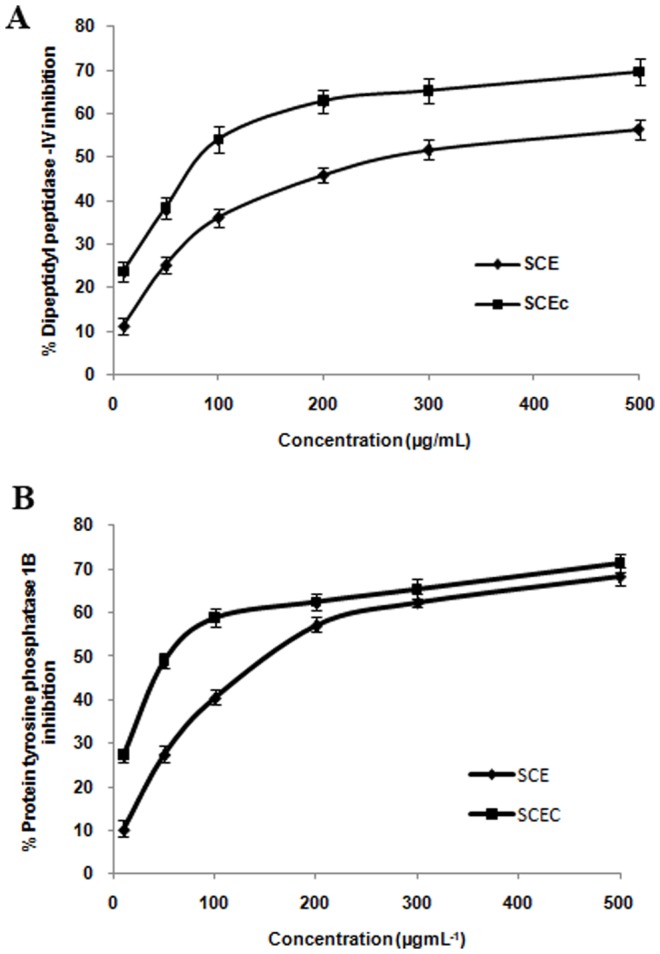
DPP-IV and PTP-1B inhibitory property of SCE and SCEC. (A) DPP-IV inhibition by SCE & SCEC. SCEC IC_50_- 87.63±1.88 µg/mL and SCE IC_50_- 269.98±2.95 µg/mL. Values are means ± SD; n = 3. (B) PTP-1B inhibitory property of SCE & SCEC. SCEC IC_50_- 55.83±1.24 µg/mL and SCE IC_50_- 159.10±1.91 µg/mL. Values are means ± SD; n = 3. SCE, *S. cochinchinensis* (SC) ethanol extract and SCEC, SC ethyl acetate fraction.

The extracts were evaluated for AG inhibition utilizing rat intestinal and yeast enzymes. SCEC, SCD and SCE showed significant yeast AG inhibition with IC_50_ values of 62.30±1.53, 71.26±1.94 and 82.07±2.10 µg/mL respectively (Fig. 1A). Rat intestinal AG inhibition (IC_50_) of the extracts was found to be 194.93±2.67 (SCEC), 143.02±2.91 (SCD) and 232.05± 3.34 µg/mL (SCE) (Fig. 1B). Acarbose showed an IC_50_ of 45±1.12 for yeast and 49.78±1.45 µg/mL for rat AG enzymes. SCEC fraction showed DPP-IV inhibition with an IC_50_ of 87.63±1.88 µg/mL while IC_50_ of SCE was 269.98±2.95 µg/mL (Fig. 2A). Standard compound diprotin A showed an IC_50_ of 1540±11.2 µg/mL. PTP-1B inhibition was noticed in SCEC fraction with an IC_50_ of 55.83 µg/mL and SCE exhibited an IC_50_ of 159.10 µg/mL (Fig. 2B). Standard was suramin (IC_50_ 14.01 µg/mL (10.8 µM)).

### SC fractions exhibited antioxidant and metal chelation potential

SCEC, SCEL and SCE showed better DPPH radical scavenging property compared to SCH and SCD (Table 2). IC_50_ of gallic acid was 6.5±0.73 µg/mL. Similarly SCEC, SCEL and SCE exhibited promising ABTS cation decolorization potential compared to SCH and SCD (Table 2). IC_50_ of the standard trolox was 5±0.51 µg/mL. SCEC, SCEL and SCE showed potent hydroxyl radical scavenging and metal chelation activity compared to SCH and SCD (Table 2). IC_50_ of catechin was 9±0.86 µg/mL and that of EDTA was 4.67±0.36 µg/mL.

**Table 2:** IC_50_ values of antioxidant (DPPH, ABTS and hydroxyl radical scavenging) and metal chelation assays.

Sl no.	Samples	IC_50_ values of DPPH radical scavenging assay in µg/mL	IC_50_ values of ABTS radical scavenging assay in µg/mL	IC_50_ values of hydroxyl radical scavenging assay in µg/mL	IC_50_ values of metal chelation activity in µg/mL
1	SCE	133.20±2.45	54.95±1.12	34.74±1.06	89.31±1.82
2	SCH	402.62±3.41	321.12±2.94	364.23±3.17	295.21±4.67
3	SCD	541.65±3.61	96.29±1.90	164.37±2.56	211.38±3.61
4	SCEC	129.43±1.84	35.72±1.02	31.64±0.98	86.49±1.76
5	SCEL	130.04±1.92	36.47±1.21	42.81±1.52	94.38±2.04

### Antiglycation property was observed in SCE, SCEC and SCEL

**Figure 3:**
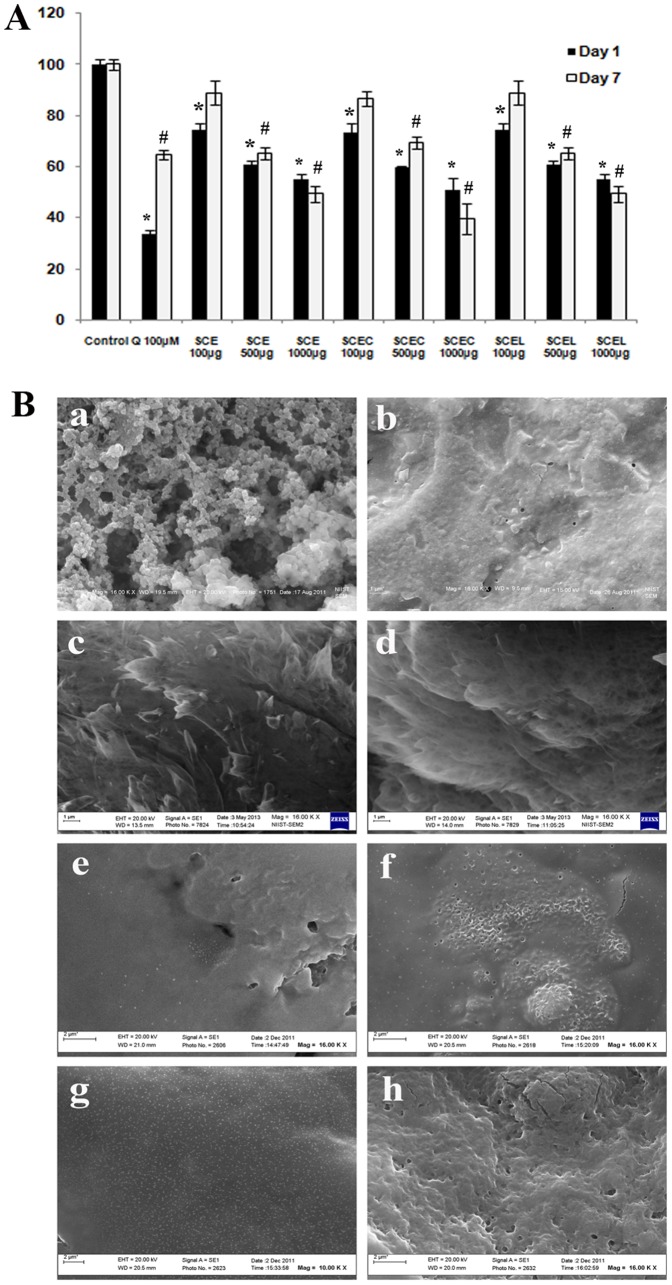
Fluorescence quantification and SEM microstructure analysis of advanced glycation end products revealed the antiglycation property of SCE SCEC and SCEL. (A) Quantification of fluorescence intensity of glycated products in presence of various concentrations of SCE, SCEC and SCEL (100, 500, 1000 µg/mL) under 2 different time intervals (day1 & day7) in terms of relative fluorescence units (RFU). Quercetin (100 µM) was used as reference compound. RFU are normalized to 100. Values are means ± SD; n = 3. *represents groups differ significantly from day 1 control group (P≤0.05) and ≠represents groups differ significantly from day 7 control group (P≤0.05). (B) Representative SEM microstructures of glycated products formed under various groups of day1 experiments (a–h), (a, control; b, quercetin 100 µM, c & d, SCE 100 µg/mL and SCE 1000 µg/mL; e & f, SCEC 100 µg/mL and SCEC 1000 µg/mL and g & h, SCEL 100 µg/mL and SCEL 1000 µg/mL. All samples were visualized at 16000× magnification.

AGEs derived from BSA was analysed using 2 methods; by RFU measurements and SEM analysis. There were 22 groups under day1 and day7 experiments. In detail, 2 untreated control groups (one each with day1 and day7 experiments), 2 quercetin treated groups (day1 and day7 experiments) and 18 extract treated groups (3 doses- 100, 500 & 1000 µg/mL of SCE, SCEC and SCEL under day1 and day7). Quercetin (100 µM) showed significant (P≤0.05) antiglycation property in RFU measurement and also in SEM analysis (Fig. 3B b). Significant decrease (P≤0.05) in fluorescence in dose dependent manner was observed in day1 and day7 experiments at 500 and 1000 µg/mL doses of three extracts, indicative of antiglycation property (Fig. 3A). SEM analysis of the microstructure of control group of day1 showed highly granular agglomeration with uneven pores and highly complex cross linking (Fig. 3B a). 500 and 1000 µg/mL doses of SCE, SCEC and SCEL reduced highly complex microstructure to simple membranous structure without any cross linking in day1 and day7 experiments (Fig. 3B c–h; day7 SEM data not shown). The SEM results were analyzed based on the previous report [21].

### Protection from hyperglycemia induced oxidative stress

**Figure 4:**
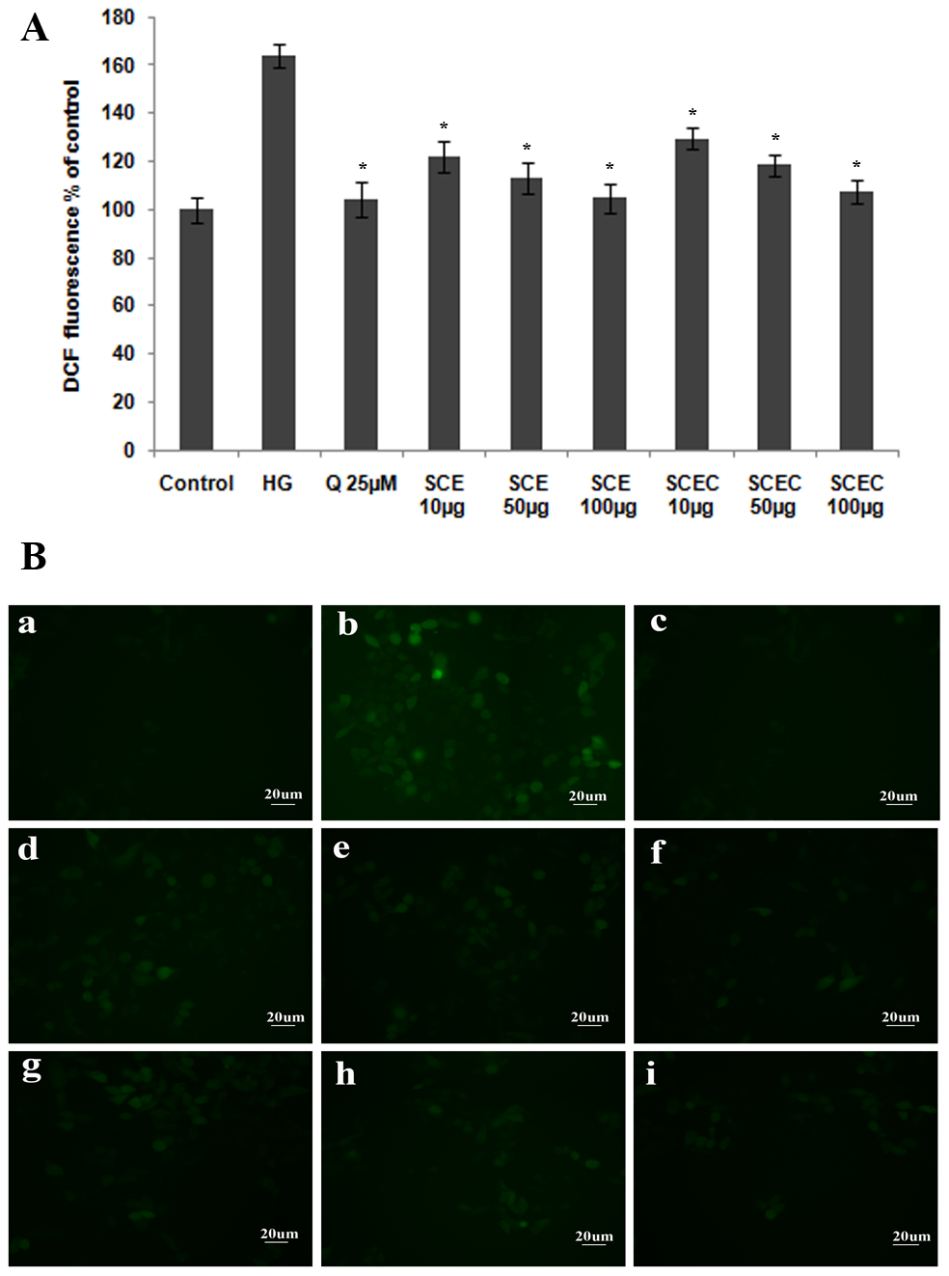
SCE and SCEC fractions protected HepG2 cells against ROS generation during hyperglycemia. Analysis of high glucose induced intracellular ROS levels in HepG2 cells by DCFDA method. Cultured HepG2 cells were treated with SCE or SCEC in the presence of high glucose (HG; 25 mM) for 24 h and then incubated with H_2_DCFDA. The results are shown as (A) the quantitative analysis of fluorescence from three independent experiments. Values are means ± SD; n = 3. *represents groups differ significantly from HG group (P≤0.05). (B) Representative microscopic scans a–i (a, vehicle control; b, high glucose (HG); c, HG+Quercetin; d–f, HG+10 µg, 50 µg and 100 µg SCE; g–i, HG+10 µg, 50 µg and 100 µg SCEC). All samples were visualized at 20× magnification.

High glucose treatment induced the generation of significant amount of ROS in HepG2 cells (64.23%; Fig. 4A and B, b), but co-treatment with SCE or SCEC significantly attenuated ROS in a dose dependent manner (P≤0.05). SCE and SCEC were selected on the basis of their potent *in vitro* antioxidant property. Results showed that 41.94, 51.28 and 59.57% decrease of ROS level with 10, 50 and 100 µg/mL SCE respectively (Fig. 4B, d–f) compared to high glucose control group. Similarly SCEC caused 34.92, 45.79 and 56.72% decrease of ROS level with 10 and 50 and 100 µg/mL dose respectively (Fig. 4B, g–i). Quercetin (25 µM) showed significant (P≤0.05) decrease (60.04%) of ROS (Fig. 4B, c). All extracts were found to be absolutely safe up to 100 µg/mL in all 5 cell lines; HepG2, L6, 3T3L1, RIN-m2F and MIN-6 (data not shown).

### Proliferation potential of pancreatic beta cells in RIN-m5F and MIN-6 cell lines

**Figure 5:**
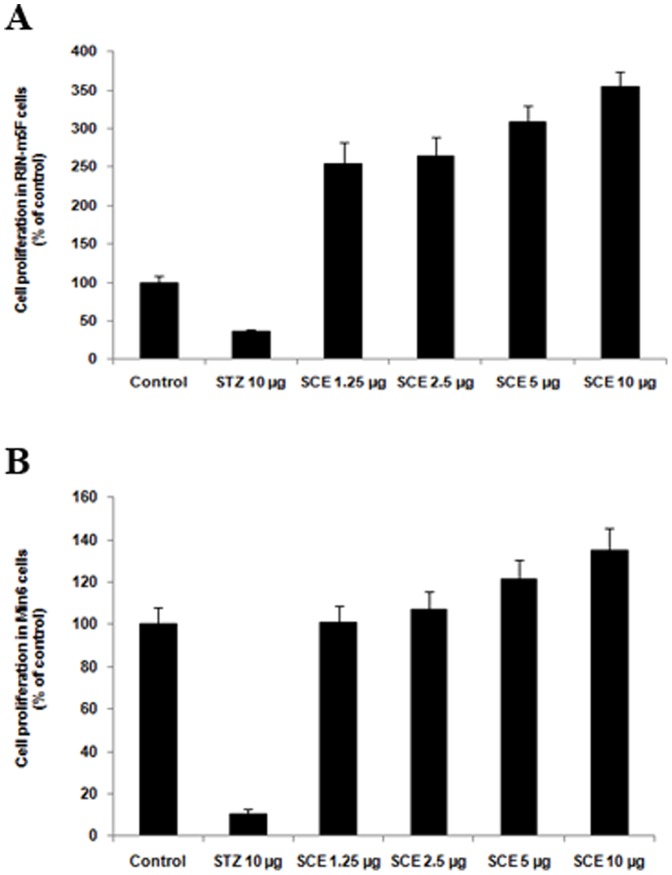
Pancreatic beta cell proliferation potential of SCE in RIN-m5F and MIN-6 cells. (A) Beta cell proliferation potential of SCE in RIN-m5F cells. (B) Beta cell proliferation potential of SCE in MIN-6 cells. Results are normalised to 100 based on control readings. Values are means ± SD; n = 3. *represents groups differ significantly from control group (P≤0.05).

Treatment with SCE (10 µg/mL) exhibited significant cell proliferation rate; 3.5 fold and 0.5 fold respectively compared to control both in RIN-m5F and MIN-6 cells (Fig. 5) and other extracts did not show any positive effect.

### Enhancement of glucose uptake in L6 myotubes

**Figure 6:**
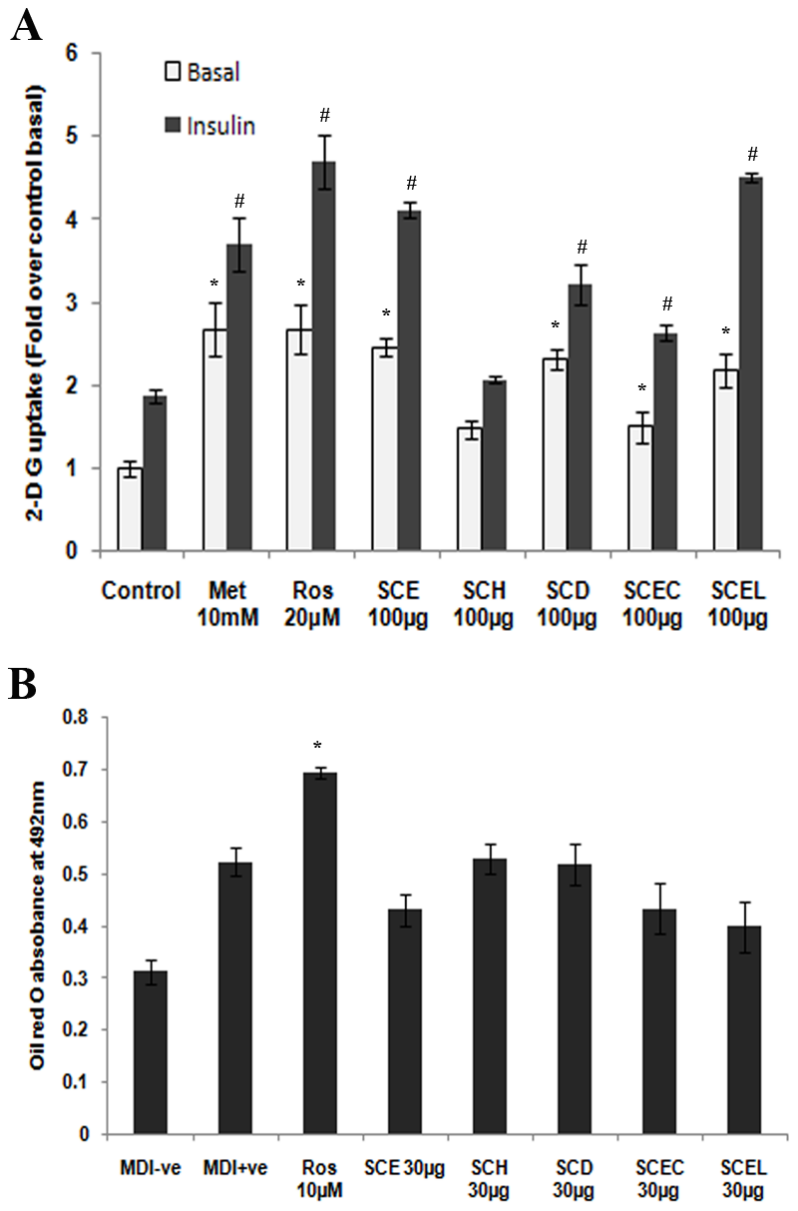
Glucose uptake and adipocyte differentiation studies in all 5 extracts. (A) 2-deoxy glucose uptake in L6 myotubes. Cells were incubated for 16 h with different extracts (100 µg/mL) or standards. After incubation myotubes were left untreated (white bars) or stimulated with 100 nM insulin (black bars) for 20 min, followed by the determination of 2-DG uptake. Results are expressed as fold stimulation over control basal. Metformin (10 mM) & rosiglitazone (20 µM) were the standards. Values are means ± SD; n = 3. *represents groups differ significantly from basal control group (P≤0.05). ≠represents groups differ significantly from insulin control group (P≤0.05). (B) Quantification of triglyceride content in differentiating 3T3-L1 adipocytes treated with different extracts (30 µg/mL) or rosiglitazone (10 µM) for 8 days by oil red O staining. Data are expressed as the means ± SD; n = 3; * represents groups differ significantly from MDI positive group (P≤0.05). MDI−ve, media without 3-isobutyl-1-methylxanthine (IBMX), dexamethasone & insulin; MDI+ve, media with IBMX, dexamethasone & insulin and Ros 10 µM, rosiglitazone 10 µM.

Pre-treatment of myotubes with SCE and its fractions for 16 h with insulin (100 nM) resulted in increase of glucose uptake in an additive manner (Fig. 6A, P≤0.05). Among various fractions studied, both SCE and SCEL exhibited better activity both in the absence and presence of insulin in a dose dependent manner (Fig. S3 in Supporting Information S1; P≤0.05). Insulin alone showed a significant increase in glucose uptake (1.9 fold of basal, P≤0.05) in L6 myotubes. Metformin and rosiglitazone were standards (Fig. 6A).

### Adipogenesis

**Figure 7:**
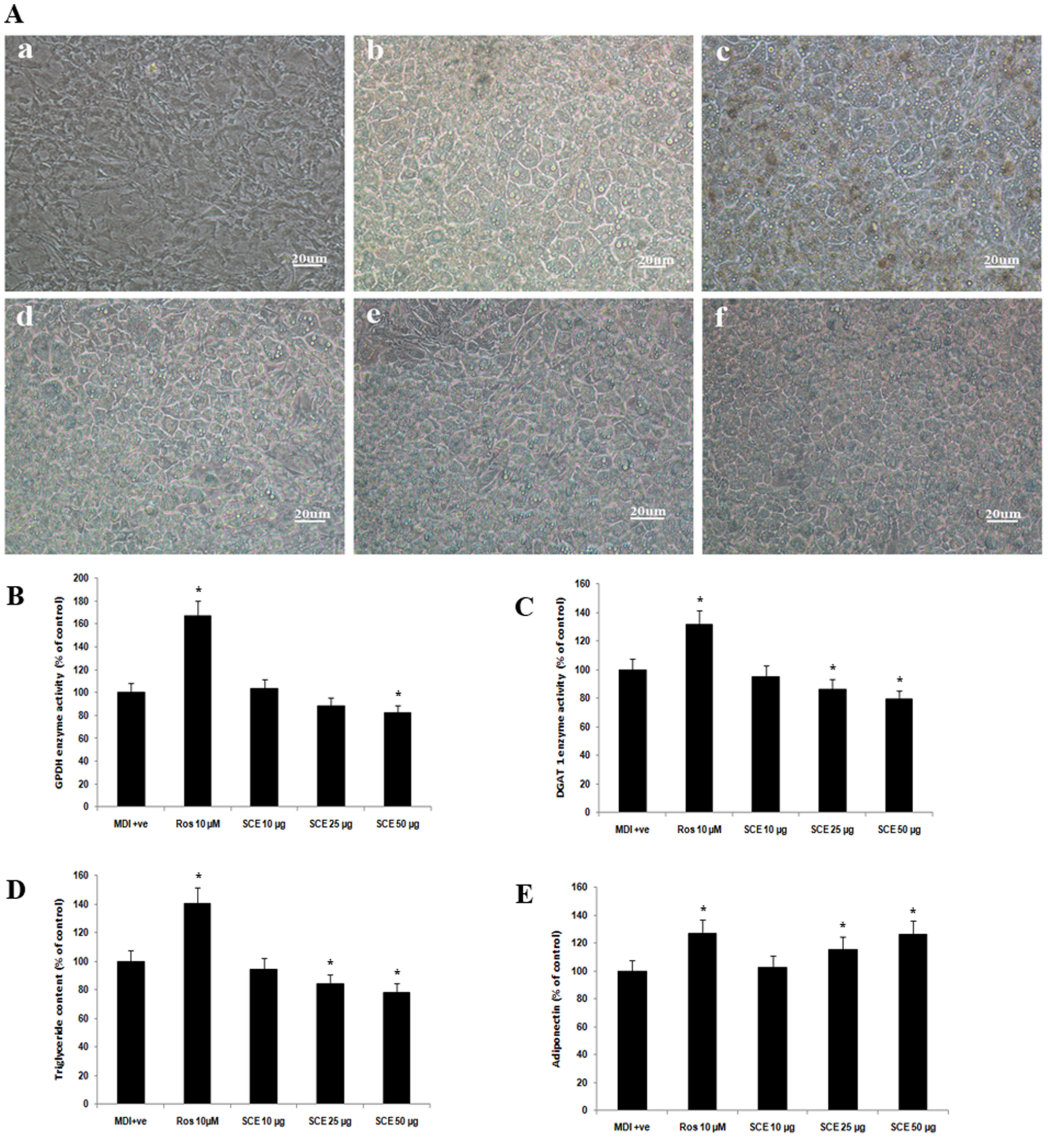
Estimation of adipogenesis in SCE tretment. (A) Cellular morphology. (Panels a–f) Micrographs (×10) showing (a) MDI negative, (b) MDI positive (vehicle control) (c) differentiating 3T3-L1 adipocytes treated for 8 days with rosiglitazone (10 µM), and (d–f) various concentrations of SCE (10, 25 and 50 µg/mL, respectively). DMSO (0.1%, vehicle) in differentiation media served as the vehicle control group i.e MDI positive. (B) Glycerol-3-phosphate dehydrogenase activity in various groups (MDI positive, rosiglitazone at 10 µM) and various concentrations of SCE (10, 25 and 50 µg/mL, respectively). (C) Diacyl glycerol -3 phosphate activity in various groups (MDI positive, rosiglitazone at 10 µM) and various concentrations of SCE (10, 25 and 50 µg/mL, respectively). (D) The triglyceride content in various groups (MDI positive, rosiglitazone at 10 µM) and various concentrations of SCE (10, 25 and 50 µg/mL, respectively). (E) Adiponectin level in various groups (MDI positive, rosiglitazone at 10 µM) and various concentrations of SCE (10, 25 and 50 µg/mL, respectively). Results are normalised to 100 based on control readings. Data are expressed as the means ± SD; n = 3. *Represents groups that differ significantly from the MDI positive (vehicle control) group (P≤0.05).

The treatment with SCE and its fractions (30 µg/mL) induced a moderate level of differentiation of 3T3-L1 preadipocytes to adipocytes, but less than rosiglitazone. This was based on the morphological observation and quantification of triglycerides by oil red O staining (Fig. 6B). SCE at 50 µg/mL dose exhibited a significant decrease in GPDH activity compared to MDI positive group (P<0.05, Fig. 7B), at the same time 25 and 50 µg/mL doses of SCE exhibited a significant decrease in DGAT1 activity and TG content compared to MDI positive group (P<0.05, Fig. 7C and D). However, adiponectin level was significantly increased by SCE treatment (25 and 50 µg/mL) compared to MDI positive group (P<0.05, Fig. 7 E). Rosiglitazone was the reference standard.

### Antihyperglycemic effect of SCE in normal and diabetic *in vivo* models

**Figure 8:**
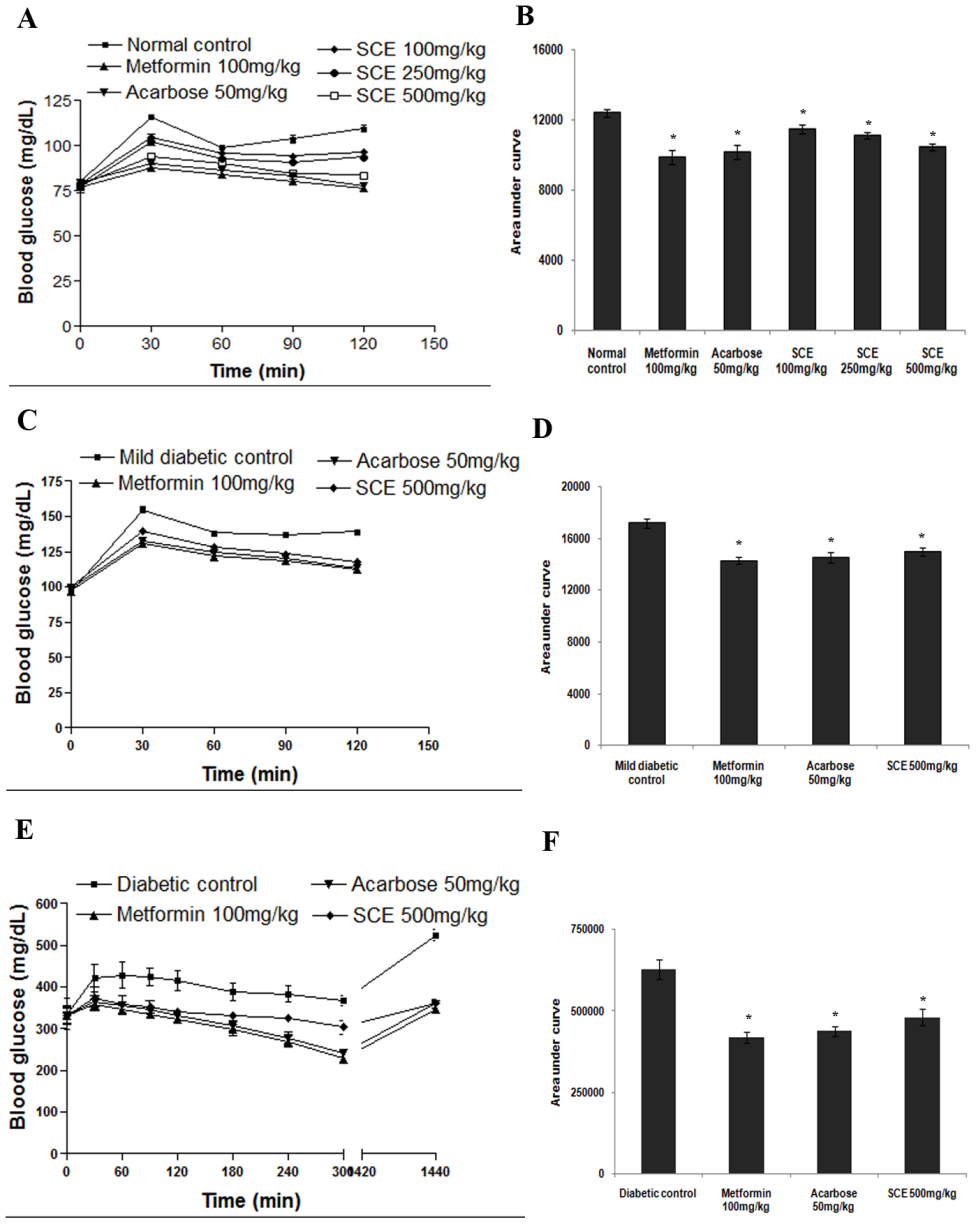
The antihyperglycemic effect of SCE in normal rats, mild diabetic rat model (SLM) and streptozotocin-induced diabetic rat model (STZ-S) after sucrose administration. (A) The glycemic response curve and (B) incremental AUC_0–120_ min in normal rats. (C) The glycemic response curve and (D) incremental AUC_0–120_ min in SLM model. (E) The glycemic response curve and (F) incremental AUC_0–1440_ min in STZ-S model. Data are expressed as the mean ± SD, n = 6. * represents groups differ significantly from control group (p<0.05). SCE, *S. cochinchinensis* (SC) ethanol extract.

In acute toxicity study, SCE did not show any observable toxic effects in behaviour or physiology of animals up to 2 g/kg bw. In normal and SLM, the rise in BGL at 30 min of oral sucrose load was significantly reduced in SCE treated group compared to control group. SCE treatment at doses of 100, 250 and 500 mg/kg bw exhibited 7.56, 10.23 and 15.53% reduction respectively in plasma glucose in normal sucrose loaded rats and 18.18 and 20.42% by acarbose and metformin treatment (Fig. 8A and B). In SLM, treatment with 500 mg/kg bw of SCE reduced the whole glycemic response by 12.88% while acarbose and metformin caused 15.73 &and 17.12% reduction respectively (Fig. 8C and D). SCE treatment (500 mg/kg bw) in STZ-S caused 23.48% improvement in blood glucose profile after 5 h of treatment and acarbose and metformin showed 30.27 and 33.18% respectively (Fig. 8E and F).

## Discussion

The pathogenesis of diabetes mellitus is complex and involves many mechanisms leading to several complications and demands a multiple therapeutic approach. Nowadays, medicinal plants have re-emerged as an effective source for the treatment of diabetes as it hold diverse group of compounds. Metformin exemplifies an efficacious oral glucose lowering agent derived from the research based on medicinal plants [34]. To date many antidiabetic medicinal plants have been reported although only a small number of these have received scientific evaluation to elucidate their mechanism of action. The World Health Organisation Expert Committee on diabetes has stressed the need of research on traditional medicine for future drugs [35]. In this study, phytochemically characterized SC was subjected to investigation on various biochemical targets relevant to diabetes like AG, glycation, DPP-IV, PTP-1B and hyperglycemia induced oxidative stress along with pancreatic beta cell proliferation, insulin dependent glucose uptake and adipogenesis using *in vivo* and *in vitro* models. Cell line based *in vitro* models are very much important in diabetic research as it is helpful to determine the mechanism of action of a plant extract with traditional use and/or human or *in vivo* data to support the antidiabetic effect [36]. In addition, the cell line based model allows the use of less amount of test material with reduced variability in results [36].

Oxidative stress due to hyperglycemia and dyslipidemia is one of the physiological parameter evident in diabetes [37]. Depletion of antioxidant level has been demonstrated in diabetic patients and extra administration of antioxidants to compensate the depletion, had helped to prevent diabetes complications [38]. Hyperglycemia induces accelerated hydroxyl radical generation and reactive oxygen species production which could represent the key event in the development of diabetic complications [39], [40]. So we had analysed the antioxidant potential and hydroxyl radical scavenging activity of various extracts and the ability of extracts to prevent ROS generation under hyperglycemia. The results revealed significant antioxidant potential of SCE, SCEC and SCEL in an *in vitro* cell free system (Table 2) and protected HepG2 cells from hyperglycemia induced oxidative stress by preventing generation of ROS (Fig. 4A & B, P≤0.05). This result is in line with the reported protective effect of SCE on hepatic oxidative stress markers in STZ diabetic animal model [41]. It has been suggested that during hyperglycemic conditions, a non-enzymatic reaction occur between proteins and monosacharides (glycation) leading to the formation of pathologically significant AGEs [42]. Due to far reaching consequences of AGEs in the body, the estimation of glycated haemoglobin (% HbA1c) has been advised by clinicians in addition to glucose in diagnosing metabolic syndrome. Biologically AGEs alter enzyme activity, modify protein and are main culprit in diabetes induced cardiomyopathy, retinopathy and neuropathy. Moreover, AGEs induce oxidative stress and vice versa [42]. So there is a tremendous interest in aniglycation agents for diabetes therapy. But as of today no specific drug is available with antiglycation potential. Our study revealed significant antiglycation activity of SCE, SCEC & SCEL (Fig. 5A and B) which could possibly one prominent mechanism of its known antidiabetic property. The two categories of antiglycation agents (AGE inhibitors and AGE breakers) act primarily as chelators by inhibiting metal-catalyzed oxidation reactions that catalyze AGE formation [43]. From the SEM microstructure analysis of AGEs, it is clear that SCE, SCEC and SCEL exhibited antiglycation via its AGE inhibitor property [44]. The *in vitro* method had shown potent metal chelation capacity of SC which may be the mechanism behind the better antiglycation potential of this plant and the reported diminished %HbA1c level in the SCE treated STZ diabetic animal model [41].

PTP-1B is an abundant and widely expressed enzyme localized in endoplasmic reticulum. Theoretically, inhibition of action of PTP-1B that terminates insulin signalling would be expected to increase insulin sensitivity [45]. SCEC and SCE showed better PTP -1B inhibitory property (Fig. 2B). DPP-IV is a serine exopeptidase which regulates the half- life of two key glucoregulatory incretin hormones like glucose dependent insulinotropic polypeptide (GIP) and glucagone like peptide-1 (GLP-1) [46]. Inhibition of DPP-IV prolongs and enhances the activity of endogenous GIP and GLP-1, which serve as important prandial stimulators of insulin secretion in response to glucose and it inhibit glucagon secretion and conserve beta cell mass [46]. SCEC & SCE exhibited moderate DPP-IV inhibitory potential (Fig. 2A).

Significantly enhanced pancreatic beta cell proliferation was noticed in RIN-m5F and MIN-6 cells by SCE treatment. This pancreatic beta cell proliferation potential of test material represent an extremely useful criteria to evaluate anti-diabetic activity, which could protect the beta cells from degeneration due to gluco-lipotoxicity during type 2 diabetes mellitus or protect from autoimmune mediated destruction as in the case of type 1 diabetes mellitus [47]. We had seen the protective property of SCE against streptozotocin induced toxicity in pancreas [41]. With this result, we strongly believe that this beneficial property contribute significantly to its antidiabetic efficiency.

Since insulin resistance is a major metabolic abnormality of type 2 diabetes, there has been considerable interest in insulin sensitizing agents to counteract insulin resistance for the treatment of this disease [3]. The result of the present study showed significant insulin dependent and independent glucose uptake proving insulin sensitizing property of SCE (Fig. 6A, P≤0.05). Further studies are required to find out the mechanism behind this effect. The peroxisome proliferator activated receptor (PPAR) gamma, the master regulator of adipogenesis is abundantly present in adipocytes which can maintain whole body insulin sensitivity and thiazolidinedione group of drugs (rosiglitazone and pioglitazone) act as PPAR modulators [48], [49]. Analysis of the effect of SCE treatment on various markers of adipogenesis such as diminished activity of GPDH and reduced TG content compared to rosiglitazone, the full PPAR gamma agonist allude partial PPAR gamma agonist property of SCE (Fig. 6B and D). Adiponecin, solely secreted from adipocytes acts as a hormone with anti-inflammatory and insulin sensitizing properties [50]. There are reports to suggest the risk of T2DM appeared to decrease monotonically with increasing adiponectin level by several mechanisms [51]. So the potential of SCE to increase adiponectin level in 3T3-L1, suggest a role in its antidiabetic property and this is the first report in this regard (Fig. 6E). But detailed study on transactivation is required to confirm this [52]. Rosiglitazone is effective insulin sensitizer [48], act through its PPAR agonism. It enhances glucose uptake and adipocyte differentiation in a variety of insulin-resistant states [53]. So rosiglitazone has been taken as positive control for both glucose uptake and adipocyte differentiation studies.

Obesity is characterized by the accumulation of triacylglycerol in adipocytes and is an important risk factor for diabetes. Diacylglycerol acyltransferase (DGAT) catalyzes the final reaction of triacylgycerol synthesis and has two isoforms DGAT1 and DGAT2. DGAT1 plays a role in VLDL synthesis; increased plasma VLDL concentrations may promote obesity and thus DGAT1 is considered a potential therapeutic target of obesity and associated complications [54]. Here, a decrease in the DGAT1 activity by the treatment of SCE was observed in the study may attribute to its potential to reduce development of obesity as well hyperglycemia induced dis/hyperlipidemia (Fig. 6C).

The postprandial hyperglycemia (PPH) became a relevant target clinically and scientifically due to the importance in cardiovascular diseases and other complications [55]. The enzyme AG, present in the intestinal brush border cells hydrolyses complex carbohydrates to simple sugars. Inhibition of AG modulate carbohydrate digestion rate and prolong overall carbohydrate digestion time, causing a reduction in the rate of glucose absorption and consequently blunting PPH and insulin levels [56]. Additional therapeutic properties of AG inhibitors include protection against pancreatic beta cell apoptosis, inhibition of attachment of macrophage to vascular endothelium and amelioration of development of atherosclerosis [57]. Initial *in vitro* screening using yeast AG is required to see whether the study material has some alpha glucosidase inhibitory property [58]. Our *in vitro* studies showed promising AG inhibitory activity against both yeast derived and rat intestinal enzymes by SCE and its fractions SCD and SCEC (Fig. 1A and B). In sucrose loaded normal and SLM models, SCE prevented acute PPH effectively compared to normal control and mild diabetic control (Fig. 8A–D, P≤0.05). This reveals the efficacy of SCE to control sucrose induced PPH significantly. This antihyperglycemic activity of SCE at 500 mg/kg bw was comparable with the existing drugs like acarbose and metformin. So we selected only 500 mg/kg dose for SLM and STZ-S studies. In streptozotocin models of diabetes, due to the destruction of pancreatic beta-cells, insulin secretion has been impaired and cause blood glucose elevation [55]. SCE treatment in STZ model resulted in attenuation of PPH, whereas diabetic control animals showed elevated blood glucose even after 5 h of sucrose load (Fig. 8E and F, P≤0.05). From this it is clear that SCE negate PPH by inhibiting AG that modify sucrose breakdown rate in small intestine in normal and diabetic rats.

Deficiency of specific vitamins and minerals play important roles in glucose metabolism and insulin signalling contribute to the development of diabetes [59]. In the present investigation, SCE was found to have high amount of calcium, moderate amount of sodium, potassium and magnesium and traces of manganese and zinc. There are also reports to link the role of these minerals in ameliorating complications arising from diabetes [59]. In addition, our TPC and TFC measurement showed the presence of high content of phenolics and flavanoids (Table 1). Accordingly, HPLC analysis revealed the presence of beta-sitosterol, phloretin 2′glucoside and oleanolic acid. All these compounds are reported to have beneficial role in diabetes as well as to attenuate diabetes induced complications via different ways: beta-sitosterol improves glucose uptake and lipid metabolism [60], [61] and alpha glucosidase inhibition [62]; phloretin enhances glucose uptake [63], [64] and oleanolic acid improves insulin response [65], [66] and possesses alpha glucosidase inhibitory property [67]. The results exhibited by SC in the present study may be due to the synergistic action of these three compounds in addition to other polyphenolic components.

## Conclusion

Overall results reveal potent antihyperglycemic activity via inhibition of alpha glucosidase and enhanced insulin sensitivity with moderate antiglycation and antioxidant potential of SC which contribute significantly to its antidiabetic property. The presence of known insulin sensitizers and AG inhibitors like phloretin-2 glucoside, oleanolic acid and beta-sitosterol in SC play an important role in these multifaceted activities of SC with respect to diabetes.

## Supporting Information

Supporting Information S1 **Figures S1–S3, Antidiabetic property of Symplocos cochinchinensis is mediated by inhibition of alpha glucosidase and enhanced insulin sensitivity.**
(DOCX)
