# Supplementary material for: Antidiabetic Property of Symplocos cochinchinensis Is Mediated by Inhibition of Alpha Glucosidase and Enhanced Insulin Sensitivity
Source: PLoS One. 2014 Sep 3;9(9):e105829. doi: 10.1371/journal.pone.0105829 (PMC4153544; doi:10.1371/journal.pone.0105829)
Supplement: Supporting Information S1 — Figures S1–S3, Antidiabetic property of Symplocos cochinchinensis is mediated by inhibition of alpha glucosidase and enhanced insulin sensitivity. (DOCX) [file pone.0105829.s001.docx]

Supporting Information S1: Figures S1-S3

**Antidiabetic property of *Symplocos cochinchinensis* is mediated by inhibition of alpha glucosidase and enhanced insulin sensitivity**

Kalathookunnel Antony Antu^1^, Mariam Philip Riya^1^, Arvind Mishra^2^, Karunakaran S Anilkumar^3^, Chandrasekharan K Chandrakanth^1^, Akhilesh K Tamrakar^4^, Arvind K Srivastava^2^, K Gopalan Raghu^1^*

^1^Agroprocessing and Natural Products Division, Council of Scientific and Industrial Research -National Institute for Interdisciplinary Science and Technology (CSIR-NIIST), Thiruvananthapuram, Kerala, India, Pin-695019.

^2^Division of Biochemistry, CSIR - Central Drug Research Institute (CSIR-CDRI), Lucknow, Uttar Pradesh, India, Pin-226001.

^3^Medicinal Chemistry Division, CSIR-CDRI, Lucknow, Uttar Pradesh, India, Pin-226001.

^4^Division of Pharmacology, CSIR-CDRI, Lucknow, Uttar Pradesh, India, Pin-226001.

^*^**For** **correspondence:** Dr. K. G. Raghu, Agroprocessing and Natural Products Division, Council of Scientific and Industrial Research - National Institute for Interdisciplinary Science and Technology (CSIR-NIIST), Thiruvanathapuram, Kerala, India, 695019.

Tel: +91 9495902522. Fax: +91 471 2491712/2491585.

e-mail: [raghukgopal2009rediffmail.com](mailto:raghukgopal2009@gmail.com)


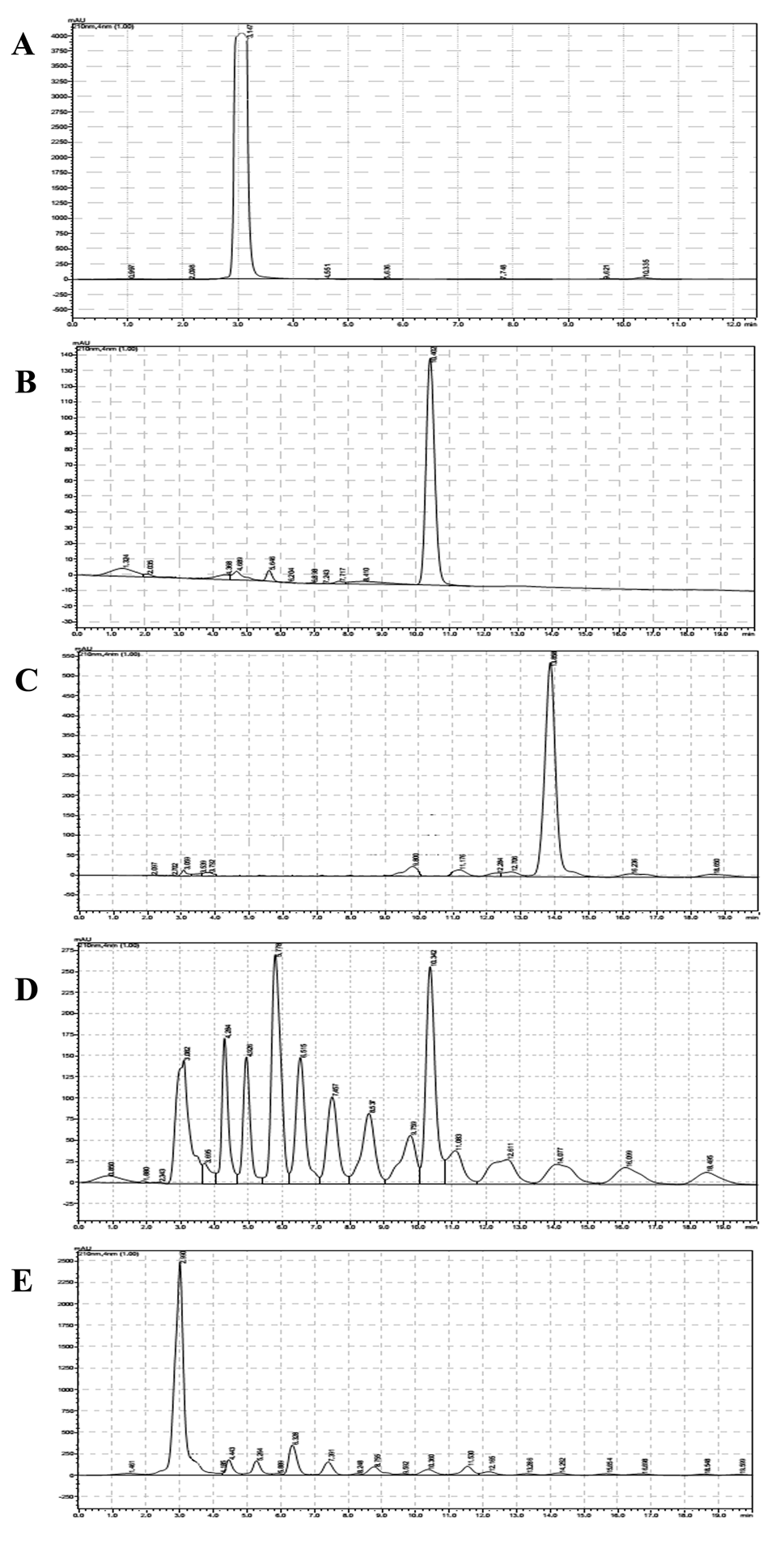


**Figure S1. HPLC data:**  HPLC chromatogram of standard compounds (A) phloretin 2’glucoside (retention time – 3.147 min), (B) beta sitosterol (retention time – 10.402 min) & (C) oleanolic acid (retention time – 13.858 min). HPLC fingerprint analysis of (D) ethanol extract of *S. cochinchinensis* (SCE) & (E) its ethyl acetate fraction (SCEC). Peaks were detected at 210 nm.

**
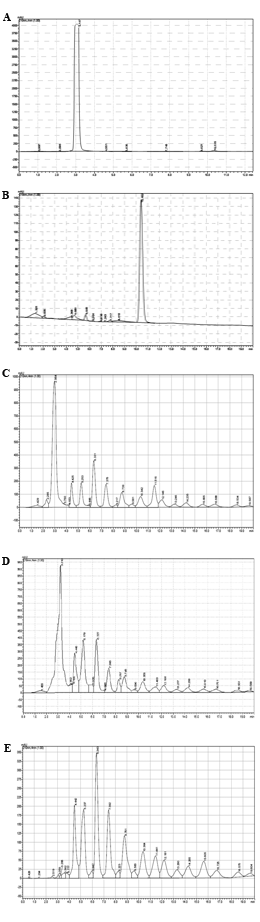
**

**Figure S2. HPLC data:**  HPLC chromatogram of standard compounds (A) phloretin 2’glucoside (retention time – 3.147 min) & (B) beta sitosterol (retention time – 10.402 min). HPLC fingerprint analysis of (C) ethanol fraction (SCEL), (D) dichloromethane fraction (SCD) & (E) hexane fraction (SCH). Peaks were detected at 210 nm.


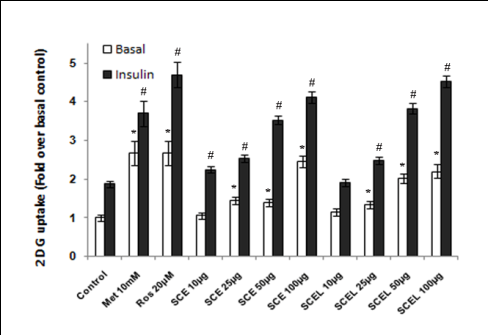


**Figure S3.** **Dose dependent** g**lucose uptake in SCE & SCEL.** (A) 2-deoxyglucose (2-DG) uptake in L6 myotubes. Cells were incubated for 16 h with different concentrations of extracts (10, 25, 50 & 100 µgmL^-1^) or standards. After incubation myotubes were left untreated (white bars) or stimulated with 100 nM insulin (black bars) for 20 min, followed by the determination of 2-DG uptake. Results are expressed as fold stimulation over control basal. Metformin (10mM) & rosiglitazone (20µM) were the standards. Values are means ± SD; n=3. *represents groups differ significantly from basal control group (P ≤ 0.05). ≠represents groups differ significantly from insulin control group (P ≤ 0.05).

**Combined Supporting Information Legend Section**

**Figure S1. HPLC data:**  HPLC chromatogram of standard compounds (A) phloretin 2’glucoside (retention time – 3.147 min), (B) beta sitosterol (retention time – 10.402 min) & (C) oleanolic acid (retention time – 13.858 min). HPLC fingerprint analysis of (D) ethanol extract of *S. cochinchinensis* (SCE) & (E) its ethyl acetate fraction (SCEC). Peaks were detected at 210 nm.

**Figure S2. HPLC data:**  HPLC chromatogram of standard compounds (A) phloretin 2’glucoside (retention time – 3.147 min) & (B) beta sitosterol (retention time – 10.402 min). HPLC fingerprint analysis of (C) ethanol fraction (SCEL), (D) dichloromethane fraction (SCD) & (E) hexane fraction (SCH). Peaks were detected at 210 nm.

**Figure S3.** **Dose dependent** g**lucose uptake in SCE & SCEL.** (A) 2-deoxyglucose (2-DG) uptake in L6 myotubes. Cells were incubated for 16 h with different concentrations of extracts (10, 25, 50 & 100 µgmL^-1^) or standards. After incubation myotubes were left untreated (white bars) or stimulated with 100 nM insulin (black bars) for 20 min, followed by the determination of 2-DG uptake. Results are expressed as fold stimulation over control basal. Metformin (10mM) & rosiglitazone (20µM) were the standards. Values are means ± SD; n=3. *represents groups differ significantly from basal control group (P ≤ 0.05). ≠represents groups differ significantly from insulin control group (P ≤ 0.05).
